# Association of *Proteus mirabilis* and *Providencia stuartii* Infections with Diabetes

**DOI:** 10.3390/medicina58020271

**Published:** 2022-02-11

**Authors:** Maria Rus, Monica Licker, Corina Musuroi, Delia Muntean, Silvana Vulpie, Oana Magiar, Teodora Sorescu, Silvia Ioana Musuroi, Adela Voinescu, Luminita Mirela Baditoiu

**Affiliations:** 1 Microbiology Department, “Victor Babes” University of Medicine and Pharmacy, No. 16 Victor Babes, 300226 Timisoara, Romania; rus.maria@umft.ro (M.R.); muntean.delia@umft.ro (D.M.); 2 Microbiology Laboratory, “Pius Brinzeu” County Clinical Emergency Hospital, No. 156 L. Rebreanu, 300723 Timisoara, Romania; vulpie.silvana@umft.ro (S.V.); oanagm@yahoo.com (O.M.); adelavoinescu@yahoo.com (A.V.); 3 Multidisciplinary Research Center of Antimicrobial Resistance, “Victor Babes” University of Medicine and Pharmacy, No. 16 Victor Babes, 300226 Timisoara, Romania; baditoiu.luminita@umft.ro; 4 Department of Diabetes, Nutrition and Metabolic Diseases, “Pius Brinzeu” Emergency Clinical County Hospital, No. 156 L. Rebreanu, 300723 Timisoara, Romania; sorescu.teodora@umft.ro; 5 Department of Diabetes, Nutrition and Metabolic Diseases, “Victor Babes” University of Medicine and Pharmacy, No. 2 Eftimie Murgu Sq, 300041 Timisoara, Romania; 6 Internal Medicine Department, Municipal Emergency Clinical Hospital, No. 5 Gheorghe Dima, 300254 Timisoara, Romania; silviamusuroi@yahoo.com; 7 Epidemiology Department, “Victor Babeş” University of Medicine and Pharmacy, No. 2 Eftimie Murgu Sq, 300041 Timisoara, Romania

**Keywords:** diabetic, non-diabetic, *P. stuartii*, *P. mirabilis*, ICU

## Abstract

*Background and Objectives*: *Proteus* and *Providencia* are related genera of opportunistic pathogens belonging to the *Morganellaceae* family, often a cause of infections in the immunocompromised hosts, such as diabetic patients. Their clinical significance has increased due to their intrinsic resistance to polymyxins, which is often associated with acquired resistance mechanisms. In this study we evaluated the infections caused by *Proteus mirabilis* and *Providencia stuartii* in two groups of patients, with diabetes (group 1) and without diabetes (group 2) admitted to the intensive care unit and surgical wards. The infections were investigated in terms of infection type, risk factors, clinical course, predictive factors for unfavourable outcomes and antibiotic resistance profile. *Materials and Methods*: An observational, retrospective, cross-sectional study was conducted, comprising all patients infected with these pathogens. Bacterial identification and antibiotic sensitivity testing were performed using the Vitek2C automated system. *Results*: Comparison of the two groups showed that the statistically significant common infectious risk factors were found less frequently among diabetic patients when compared with non-diabetic patients, and that antimicrobial resistance was significantly lower in the diabetic patient group. However, survival rates did not differ between the two groups, drawing attention to the implications of diabetes as comorbidity. Additionally, with regard to the antibiotic resistance profile, 38.89% of *P. stuartii* strains isolated from diabetic patients belonged to the difficult-to-treat (DTR) phenotype, contributing to the severity of these infections compared with those caused by *P. mirabilis*, of which 32% were wild type strains and 0% were DTR phenotype. The DTR/extended spectrum beta-lactamase producing *P. stuartii* isolates more than doubled the risk of mortality, while the presence of nasogastric nutrition tripled the risk. *Conclusions*: *P. stuartii* infections that occurred in diabetic patients proved to be more difficult to treat, the majority of them being healthcare-associated bacteremias.

## 1. Introduction

People with diabetes are more prone to develop infections compared with the general population [1,2]. The increased susceptibility to infections of diabetic patients may be partially explained by some defects in host immune defence mechanisms, such as the impairment of neutrophil function, affecting endothelial adhesion, chemotaxis, phagocytosis and “natural killer” activity [3,4]. In type 1 diabetes, more alterations occur, such as the decrease in the number of T lymphocytes, especially CD 4 (T helper), the reduction of the CD 4/CD 8 ratio and of the IgG and IgA production and also quantitative and qualitative alteration of serum complement components [5]. In addition to the generalized immune system disorders, chronic complications of diabetes, such as microangiopathy and macrovascular disease, represent an additional risk for infection, leading to local vascular impairment and impaired wound healing [3,5]. Peripheral sensory neuropathy may lead to unawareness of lower limb trauma and increased infection risk [6]. Incomplete bladder emptying due to autonomic neuropathy, or the presence of glycosuria allow urinary colonization by microorganisms [3].

The risk of skin and mucosal infections (due to more abundant colonization with staphylococci and *Candida* spp), diabetic foot infections (DFI) (ranging from cellulitis to soft tissue necrosis and sometimes osteomyelitis) and an increased frequency of respiratory and urinary tract infections (UTIs) are well known, representing a clinical and therapeutic challenge [7,8].

Asymptomatic bacteriuria, three-times more common in diabetic than in non-diabetic women [9], occurs mainly secondary to neuropathy (leading to an increased volume of post-void residual urine) [10] and to increased adhesion of microorganisms to uroepithelial cells. The risk of cystitis, pyelonephritis, perinephritis and papillary necrosis is also increased, potentially with significant local and systemic complications.

Respiratory tract infections may be severe, with a mortality from pneumonia four-times higher in diabetics versus non-diabetics. A patient with diabetes is also six to seven-times more likely to develop pulmonary tuberculosis, often oligo- or asymptomatic, due to cellular immune system impairment, with the possibility of unfavourable clinical progression and increased rates of mortality [5].

Increased susceptibility to infections represents an additional risk for healthcare associated infections (HCAI) in hospitalized diabetic patients. In the United States, 11% of patients undergoing lower limb bypass develop surgical site infections (SSIs), diabetes being considered a significant risk factor (OR = 1.5; 95% CI 1.2–1.8) [11]. Diabetes and postoperative hyperglycaemia were identified as independent risk factors for SSIs associated with multiple surgical procedures [12].

The risk of graft infection is higher in patients with diabetes (HR = 4.6; 95% CI 1.5–14.3) and graft infection is a risk factor for major amputation of the lower extremities (HR, 9.8; 95% CI 3.5–27.1). It has been shown that 29% of patients with an infected graft suffered a major amputation 1 year after an intervention [13].

Moreover, there is an increased risk of sepsis in diabetic patients compared with non-diabetic patients (RR = 2.45; 95% CI 2.23–2.68), with a similarly increased risk for SSIs (RR = 2.02, 95% CI 1.80–2.27) and osteomyelitis (RR = 4.39; 95% CI 3.80–5.06) [11]. With regard to the aetiology of infections, diabetes increases the prevalence of infections from less common organisms such as *Klebsiella* spp., *Pseudomonas* spp., *Proteus mirabilis*, enterococci and fungi [14].

*Proteus* and *Providencia* are related genera of Gram-negative bacilli, opportunistic pathogens belonging to the order *Enterobacterales*, in the *Morganellaceae* family, and the *Proteeae* group, which often cause HCAIs in immunocompromised hosts. Their clinical significance has increased due to their intrinsic resistance to polymyxins (considered reserve antibiotics), which is associated with acquired resistance mechanisms [15].

*Providencia stuartii* was isolated mainly in UTIs, being strongly associated with biofilm formation in catheterised patients [16]. In addition, the synthesis of broad-spectrum beta-lactamases (ESBL) and metallo-beta-lactamases, as well as the association of other genetic resistance mechanisms make it a difficult-to-treat (DTR) microorganism [17]. In a recent study (2021) regarding the *Morganellaceae* species, represented mainly by *P. stuartii* and *P. mirabilis*, excess mortality in the DTR subsample was 16.37% compared with the non-DTR one [18].

Over the last decade, in several Romanian hospitals, including our hospital, an alarming increase in the incidence of carbapenem resistant *Proteeae* in samples from patients admitted to high-risk wards was reported [15,19,20,21,22,23].

The aim of this study was to assess *P. mirabilis* and *P. stuartii* infections in diabetic and non-diabetic patients admitted to the intensive care unit (ICU) and surgical wards, with regard to risk factors, type of infection, clinical progression, and predictive factors for unfavourable outcomes and resistance phenotypes, respectively.

## 2. Materials and Methods

An observational, retrospective, cross-sectional study was conducted at the “Pius Brinzeu” Emergency Clinical County Hospital Timișoara between July 2017 and April 2019. The study comprised all patients admitted to the ICU and to surgical wards, from whom *P. mirabilis* and *P. stuartii* were isolated.

Patients under the age of 18 were excluded, as well as those admitted to the ICU and to surgical wards for less than 24 h. Those re-admitted were registered for a second time in the database only if they presented a strain of a species or a new resistance phenotype different from the one previously present.

In order to highlight the impact of diabetes as a comorbidity, the sample of patients was subdivided into two groups:

Group 1 (G1)—All patients who developed infections with pathogens belonging to the two species identified above and who had been previously diagnosed with type 1 or type 2 diabetes mellitus (DM); latent autoimmune diabetes of the adult (LADA) or maturity-onset diabetes of the young (MODY); patients with prediabetic conditions (impaired glucose tolerance/impaired fasting glucose), gestational diabetes, or with other specific types of diabetes secondary to exocrine pancreatic diseases (cystic fibrosis), endocrine pathology (Cushing’s syndrome, hyperthyroidism), or genetic pathology (Down syndrome), as well as those with hyperglycaemia secondary to glucocorticoid therapy, thiazide diuretics, etc. were not included in this group.

Group 2 (G2)—All patients infected with pathogens belonging to the two species identified above, who were hospitalized in the same wards (ICU or surgical) during the same period of time, but who did not have diabetes as a comorbidity.

HCAI are defined according to the criteria of the European Union (EU) decision 2018/945 on Communicable Diseases and Related Special Health Issues to Be Covered by Epidemiological Surveillance [24]. The risk associated with medical devices such as intubation probe, central vascular catheter and urinary catheter was also investigated.

The data collection complied with the requirements of EU Regulation No. 679/2016 on the protection of individuals with regard to the processing of personal data and the free movement of such data. The approval of the Ethics Committee of the ”Pius Brinzeu” Clinical Emergency County Hospital Timișoara was requested and received (No. 149/06.02.2019).

Microbiological method. The primary culture was performed according to the Bacteriology Laboratory working protocol. Blood (from positive Bactec blood culture bottles), catheter tips, wound swabs, urine, bronchial aspirates) were cultured on solid media (Columbia blood agar Sanimed International Impex) and chromogenic agar (UTI, Sanimed International Impex). Bacterial identification and antibiotic sensitivity testing were performed using the Vitek2C automated system, according to the Clinical Laboratory Standards Institute (CLSI) 2017–2018 standard. *Escherichia coli* ATCC 25922 and *Klebsiella pneumoniae* ATCC BAA-1705 reference strains were used. The carbapenem-resistant phenotype was identified using the KPC/MBL kit and OXA-48 (Rosco Diagnostica).

Antibiotic resistance phenotypes were defined as follows:

Multidrug-resistant (MDR)—Resistance to at least one antibiotic from three or more classes of antibiotics active for a given species [25,26];

Extensively drug resistant (XDR)—Resistance to at least one agent in all but one or two antimicrobial categories [25,26];

Pan-drug resistant (PDR)—Resistance to all agents in all active antimicrobial categories for a particular species [25,26];

Wild type/susceptible (WT)—Strains with natural sensitivity, characteristic of that species [27];

Difficult-to-treat resistance (DTR)—Resistance to all first-line antimicrobials, represented by carbapenems (imipenem, meropenem and ertapenem/doripenem), broad-spectrum cephalosporins (the ones relevant for those pathogens) and fluoroquinolones (ciprofloxacin, levofloxacin and moxifloxacin) [28].

Statistical analysis. Data analysis was performed using IBM SPSS Statistics 20 (SPSS Inc., Chicago, Il). Continuous variables were characterized by the median and interquartile ranges (IQR) and categorical variables were characterized by value and percentage. Data distribution testing was performed using the Kolmogorov–Smirnov test. The numerical variables were compared using the Mann–Whitney U test and the nominal ones using the chi^2^ test. The bivariate correlation was established by applying the Spearman correlation coefficient. The independent variables that were statistically significant in the univariate analysis were further investigated by logistic regression, the model being chosen according to the Nagelkerke R^2^ coefficient and according to the test for assessing deviation from the theoretical model of Hosmer and Lemeshow. Predictors of mortality were investigated using Cox proportional hazards regression models. The Kaplan–Meier method with the log-rank (Mantel-Cox) test was used to compare survival rates between the two samples. All statistical tests were calculated with 2 extremities and the threshold of statistical significance was considered *p* ≤ 0.05.

## 3. Results

A total of 339 patients, from whom 245 unduplicated strains of *P. mirabilis* and 94 strains of *P. stuartii* were isolated, were identified in the ICU and surgical wards during the study period.

**Figure 1:**
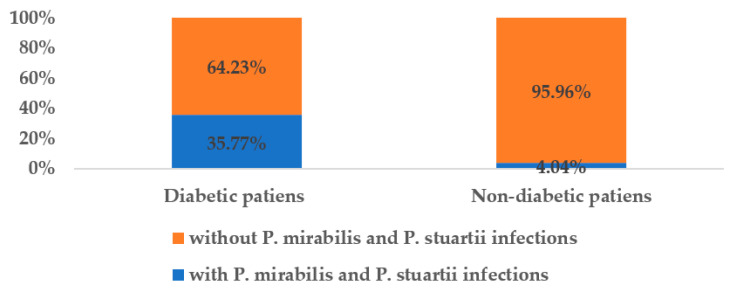
The share of infected patients in the total number of diabetic/non-diabetic patients.

Of the 339 patients, 93 (27.43%) had diabetes and, out of these, the majority (80.65%) had *P. mirabilis* infections, and only 19.35% had *P. stuartii* infections. Patients infected with *Proteeae* species represented 35.77% (over one third) of all diabetic patients admitted to the ICU and surgical wards, while only 4.04% of all non-diabetic patients became infected with the two species mentioned above (Figure 1).

The majority (62.36%) of the diabetic patients infected with *Proteeae*, came from the surgical wards (due to more frequent amputations, abscess drainage and other DFI or SSI) while in the group of non-diabetics, the majority (60.16%) came from the ICU (following complications after orthopaedic, thoracic, neuro-surgical interventions).

**Figure 2:**
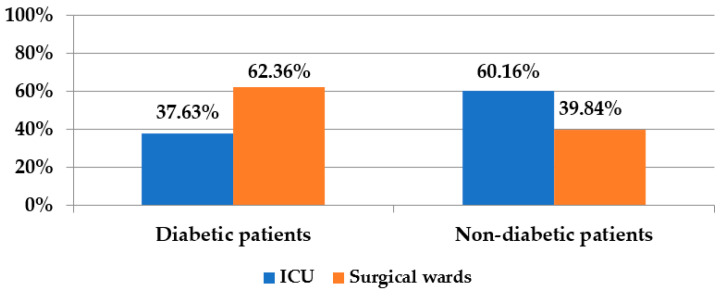
Distribution of diabetic and non-diabetic patients according to the profile of the ward.

**Figure 3:**
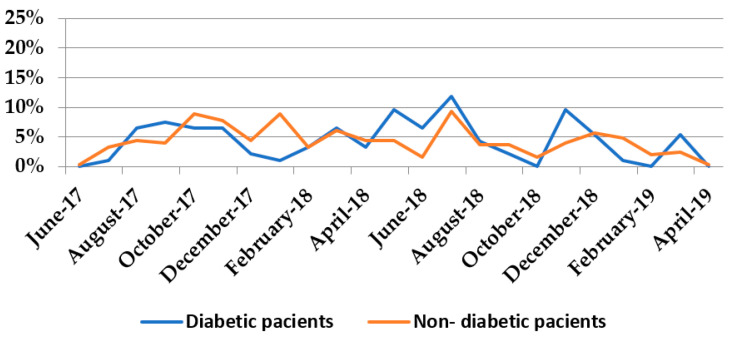
Distribution of infections with *P. mirabilis* and *P. stuartii* in the studied time interval.

The distribution of patients according to the profile of the ward where they were treated is given in Figure 2:

The highest number of infections in both diabetic and non-diabetic patients was recorded in July 2018, which shows that there are predisposing factors for *Proteeae* infections in both DM and in non-DM groups, related to environmental conditions such as temperature (July being a warm month). (Figure 3). The Spearman correlation coefficient between the monthly distribution of infections among diabetic and non-diabetic patients was 0.519, being statistically significant (*p* = 0.011).

Comparison of the two groups (Table 1) shows that the statistically significant common infectious risk factors were found less frequently among diabetic compared to non-diabetic patients. Logistic regression identified only *P. mirabilis* infections (HR = 6.08 [1.18–31.45], *p* = 0.031) and wound infections (HR = 8.43 [1.19–59.65], *p* = 0.033) as independent risk factors for diabetic patients. In contrast, diabetes as a comorbidity recorded protective values against infections with aminoglycoside-resistant (HR = 0.21 [0.05–0.82], *p* = 0.025), carbapenem resistant (HR = 0.03 [0.002–0.60], *p* = 0.021) and fluoroquinolone-resistant strains (HR = 0.19 [0.04–0.83], *p* = 0.027), respectively.

All these mean that one third of the DM group, especially patients hospitalized in surgical wards, were infected with *Proteeae*, the predominant being sensitive *P. mirabilis* strains in contrast to the non-DM group, most of them from ICU, usually with HCAI produced by MDR *P. stuartii* strains.

**Figure 4:**
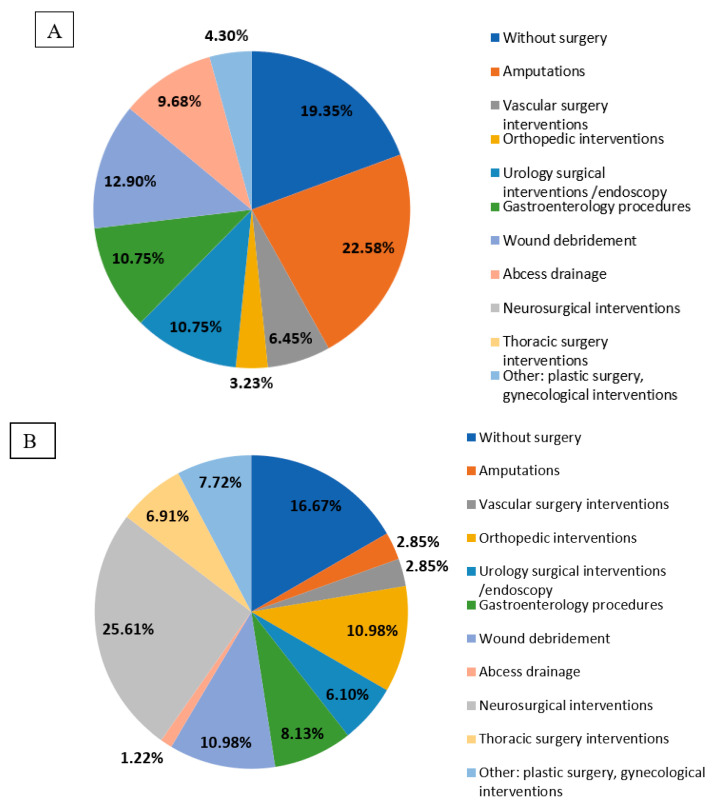
Distribution of surgical interventions/procedures performed in diabetic (**A**) and non-diabetic (**B**) patients.

The differences regarding risk factors arose from the different therapeutic interventions. Thus, diabetic patients suffered significantly more frequent amputations (*p* < 0.001) and abscess drainage (*p* < 0.001), while non-diabetic patients underwent orthopaedic (*p* = 0.025), thoracic (*p* = 0.004) and neurosurgical interventions (*p* < 0.001) more frequently. (Figure 4). These findings could be the consequence of angiopathy and neuropathy (in the diabetic patient group) and the increased frequency of traumatic pathology in adulthood, as well as the fact that these second group of surgeries were often the cause of prolonged admission, ICU transfer and acquisition of hospital acquired pathogens (in the non-diabetic patients group).

**Figure 5:**
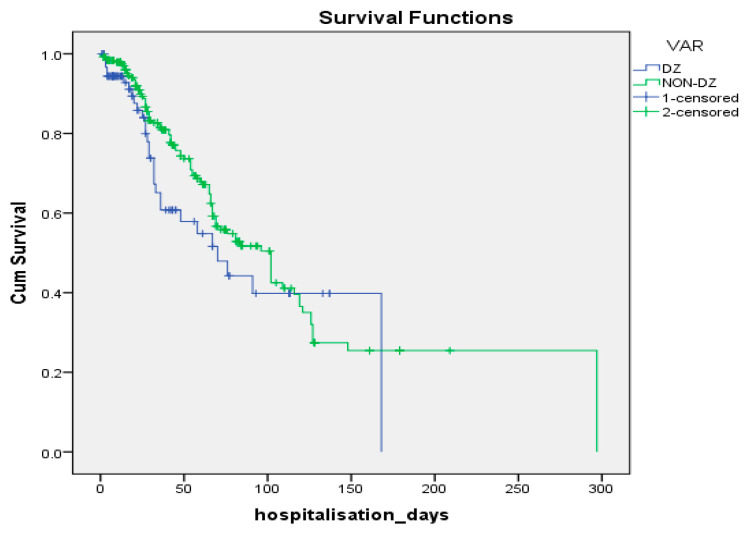
Kaplan–Meier curves of the subjects in the two samples.

Although the complexity of therapeutic interventions and risk factors were significantly more pronounced among G2 patients, survival rates did not differ between the two groups (log-rank Mantel-Cox test *p* = 0.163) (Figure 5).

Cox regression identified the following factors associated with risk of in-hospital death:

- Age HR = 1.029 (1.011–1.047), *p* = 0.001;
- Vasopressor therapy HR = 1.851 (1.080–3.171), *p* = 0.025;
- Nasogastric nutrition HR = 3.137 (1.292–7.618), *p* = 0.012;
- Bacterial species HR = 2.021 (1.145–3.565), *p* = 0.015;
- DTR strains HR = 2.726 (1.245–5.965), *p* = 0.012;
- ESBL strains HR = 2.175 (1.070–4.423), *p* = 0.032;
- Duration of antibiotic therapy HR = 0.971 (0.959–0.984), *p* < 0.001;
- Duration of urinary catheterization HR = 0.974 (0.955–0.993), *p* = 0.007.

Diabetic patients infected with *P. stuartii* had a significantly longer duration of central venous and urinary catheters in situ, a longer period of mechanical ventilation and a higher rate of antimicrobial resistance, which may be explained by the fact that 72.22% of infections were HCAI. 66.67% of *P. stuartii* infections were blood stream infections, while 64.00% of *P. mirabilis* infections were wound infections, two thirds of them being community-acquired (Table 2).

## 4. Discussion

Diabetic foot is one of the most common complications of diabetes, and also a major public health concern worldwide, with an incidence of up to 10% among this category of patients. Furthermore, treatment often involves partial or total amputation of the affected lower limb [29]. The most commonly isolated pathogens in a study of 440 patients with DFI were Gram-negative aerobic bacteria (51.2% of the total isolates). Along with the isolates of *Proteus* spp. (47; 12.0%), *P. aeruginosa* (135; 34%), *E. coli* (46; 12%), *K. pneumoniae* (36; 9.0%) and *Enterobacter* spp. (34; 8.5%) were identified, highlighting that DFI were mainly polymicrobial and that most of the etiological agents were MDR [30].

Related to the above, in a prospective study which enrolled 216 diabetic patients with DFI, Saseedharan pointed out that the identified pathogens were most frequently Gram-negatives (58.5%), with a frequency of 5.45% for *P. mirabilis* strains, along with *E. coli* (20.6%), *K. pneumoniae* (16.36%) and *C. koseri* (6.06%) [31].

Another recent review of the diversity of microorganisms involved in DFI identified the *Enterobacterales (E. coli, K. pneumoniae, M. morganii* and *P. mirabilis)* as the largest group of Gram-negative pathogens involved [32].

With regard to the relationship between diabetes and SSIs, Martin showed that this association was significantly higher for cardiac surgery compared to other types of surgical interventions, and that diabetes was identified as an independent risk factor for multiple types of surgical procedures [12]. Furthermore, Jeffrey showed that the rate of graft infection involving femoral artery was significantly more frequent in patients with diabetes compared with patients without this pathology (6.5% vs. 1.9%; *p* < 0.01), having a total graft infection frequency of 3.8% [13].

In this study, we aimed to evaluate the infections caused by *P. mirabilis* and *P. stuartii* in diabetic versus non-diabetic patients admitted to high-risk wards of our hospital from the perspective of risk factors, type of infections, clinical evolution, and predictive factors, respectively, of resistance phenotypes.

From the beginning, it was observed that diabetes, as a comorbidity, was an important risk factor for infections with the two species of Proteeae (OR = 13.24 [9.87–17.76], *p* < 0.001). The diabetic patients came mainly from surgical wards, while those without this pathology were mostly admitted to the ICU (*p* < 0.001), thus involving a greater degree of complexity and invasiveness of the diagnostic and therapeutic procedures.

There were significant differences in the nature of the surgical interventions between the two groups of patients: in G1, amputations (22.58%) and wound debridements (12.90%) predominated, followed by urological and gastroenterological procedures (10.75%), whilst in G2, neurosurgical interventions prevailed (25.61%), followed by orthopaedic procedures and wound debridements (10.98% each). Thus, one third of diabetic patients underwent surgical procedures directly related to the complications of diabetes, while approximately a quarter of non-diabetic patients had neurosurgical interventions which were associated with altered consciousness (also highlighted by the lower average value on the Glasgow Coma Scale), the need for prolonged mechanical ventilation, tracheostomy and increased duration of catheterization.

The fact that the peak monthly frequency of infections caused by these two *Proteeae* species is the same in both groups, as well as the direct, positive, moderate, and statistically significant correlation (rho = 0.519, *p* = 0.011) between the two groups, argue for the contribution of exogenous microbial flora transmission in the hospital environment. In fact, as expected, the percentage of HCAI is almost double in G2 compared with G1 (59.76% vs. 32.96%, *p* < 0.001), due to the higher percentage of iatrogenic factors. It also raises the issue of high ambient temperature as a predisposing factor for infections with the two species. The median age of patients with Proteeae infections in our study was 65.00 years [56.00–84.00] for diabetic patients and 61.00 years [48.00–69.00] for non-diabetic patients, lower than the median age of 69.6 years (IQR 41.6–81.8) recorded by Laupland (2007) in his study [33]. The author showed a significant relationship between age, sex and the incidence of Proteeae identification, with dramatically increasing rates over the age of 60 and with a twofold increased risk in women compared with men.

The predominantly community-acquired nature of infections and the known increased susceptibility to infections of diabetic patients explain the lower frequency of isolates resistant to the main classes of antimicrobial agents, as well as the results obtained by logistic regression in our study.

Diabetic patients had *P. mirabilis* infections (HR = 6.08 [1.18–31.45], *p* = 0.031) and wound infections (HR = 8.43 [1.19–59.65], *p* = 0.033) as independent risk factors. It is important to note that these two risk factors usually coexist in studies that report the presence of *Proteus* spp., especially in DFI.

UTI is another common pathology of the diabetic patient, the main risk factors being poor glycemic control, duration of diabetes, diabetic microangiopathy, impaired leukocyte function, recurrent vaginitis and anatomical and functional abnormalities of the urinary tract [8,9,10,34,35].

In the study conducted by Kumar et al., 49.01% of UTIs in diabetic patients were caused by *E. coli*, 11.07% by *K. pneumoniae* and 8.3% by *P. mirabilis*. *P. aeruginosa*, with the *A. calcoaceticus-baumannii* complex ranked 4th and 5th in prevalence [36].

Moreover, Gautam et al. showed in their study that UTIs caused by *C. albicans* and *P. mirabilis* coinfection were more frequent in the diabetic patient group compared with the non-diabetic group (*p* = 0.002 and *p* = 0.02, respectively) and that 50% of the *P. mirabilis* isolates were MDR [37].

*P. stuartii* is more commonly isolated from UTIs in patients with long-term indwelling urinary catheters than in non-catheterized patients [38,39,40], persistent bacterial adherence being mediated by type 3 fimbriae [41].

A study conducted in a Korean hospital which investigated the clinical features and antibiotic susceptibility of *Providencia* species in 14 cases of bacteremia revealed that UTIs were the most common source (35.7%), and that the *Providencia* spp. showed resistance to tobramycin but were susceptible to amikacin and isepamicin. Susceptibility to ciprofloxacin was seen in 50% of the isolates, higher than in the present study [42].

Another study performed in an Italian university hospital which investigated *P. stuartii* infections over a four-year period found that 52% of the isolated strains were ESBL-producers, all of them being MDR [43]. Sabri et al. [40] also identified the broad-spectrum antimicrobial resistance of this organism. Out of the 32 samples of *P. stuartii* isolated mostly from catheterized urine samples, 53.1% were ESBL-MDR isolates and 94% of them were AmpC producers.

In the present study, all patients with *P. stuartii* UTIs had a long-term urinary catheter with an average duration of 48.75 days, while in the case of *P. mirabillis* UTIs only 43.33% of patients were catheterized, with an average duration of bladder catheterization of 49.23 days.

In our study, *P. mirabilis* and *P. stuartii* isolates were responsible for 10.75% of the UTIs in diabetic patients and for 13.41% of the UTIs in the non-diabetic group. Of these two species, *P. mirabilis* was predominant in UTIs of both diabetic (96%) and non-diabetic (63%) patients, highlighting that, in the non-diabetic group, *P. stuartii* was involved in a third of these infections.

The importance of diabetes as a comorbidity is reflected in the survival analysis, although G1 subjects had fewer risk factors for infection and the strains were less resistant and the length of hospitalization/catheterization was shorter, survival did not differ when compared with G2 patients (log-rank test *p* = 0.163). Multivariate Cox regression analysis revealed the following factors associated with risk of in-hospital death:

- A 1-day increase in the duration of urinary catheterization decreased the risk of death by 2.6%;
- A 1-day increase in the duration of antimicrobial therapy decreased the risk of death by 2.9%;
- Increasing age by 1 unit increased the risk of death by 2.9%;
- The presence of vasopressor therapy increased the risk of death by 85.1%;
- Infection with *P. stuartii* increased the risk of death by 102.1%;
- Infection with ESBL strains increased the risk of death by 117.5%;
- Infection with DTR strains increased the risk of death by 172.6%;
- The presence of nasogastric nutrition increased the risk of death by 213.7%.

Regarding bloodstream infections caused by *P. stuartii*, Hayakawa et al. [44] showed that the frequent use of colistin and tigecycline increased the risk of infections with these species due to selective antimicrobial pressure, the microorganism being intrinsically resistant to these antibiotics, being well known *P. stuartii* intrinsic resistance to colistin, tigecycline, and reduced sensitivity to aminopenicillins, the first generation of cephalosporins and aminoglycosides [45].

The comparison presented in Table 2 highlights the severity of infections caused by *P. stuartii* compared to those caused by *P. mirabilis*, with significant differences regarding risk factors, antimicrobial resistance, prolonged duration of catheterization and treatment difficulties, due to the significantly higher number of antimicrobial agents used. A total of 66.66% of the infections caused by *P. stuartii* were blood stream infections, either primary or secondary, resulting from the spreading of an infection from a different location. A total of 38.89% of *P. stuartii* strains belonged to the DTR phenotype, which contributed to the severity of these infections compared with those caused by *P. mirabilis*, 32% of which were WT strains while 0% were DTR phenotype.

Our study has several limitations: It is an unicentric study, with a retrospective design, which compares diabetic patients to patients without this pathology, with no stratification according to glycaemic control. The cross-sectional model allowed the samples to be matched only in terms of the location and duration of hospitalization. The variables regarding the risk factors (days of hospitalization/antibiotic therapy/catheterization) represent total values and not the values strictly related to the period prior to the onset of infection, in order to more accurately quantify the impact of HCAI. The unequal size of the two samples of infected diabetic patients influenced the difference in mortality rates during hospitalization, which only reached the statistical significance threshold (*p* = 0.054), although the percentage was almost double for *P. stuartii* infections. However, the manuscript presents preliminary data, which needs to be confirmed by a more significant population, the importance of infectious pathology produced by Proteeae species and risk factors involved in diabetic patients requiring new research directions.

## 5. Conclusions

Although diabetic patients had fewer risk factors for infection and their antimicrobial resistance was significantly lower, survival did not differ from non-diabetic patients, drawing attention to the implications of diabetes as a comorbidity. The DTR/ESBL *P. stuartii* isolates had the effect of doubling, at the very least, the risk of death, while the presence of nasogastric nutrition tripled it.

*P. stuartii* infections proved to be more difficult to treat in diabetic patients, most of them being healthcare-associated bacteremias which require improved infection control measures, ranging from patient isolation, decontamination, and sterilization of equipment to staff training, in order to prevent complex indirect transmission of this pathogen.

## Figures and Tables

**Table 1:** Variables with statistical significance in univariate analysis.

Variable	G1-Diabetic Patients N1 = 93	G2-Non-Diabetic Patients N2 = 246	*p* Value	OR [95% CI]
Age [median, IQR]	65 (28)	61 (21)	0.002	0.97 [0.95–0.99]
Days of hospitalization [median, IQR]	23 (35)	42 (55)	<0.001	1.01 [1.00–1.02]
Days of antibiotic therapy [median, IQR]	12 (36)	23 (41)	<0.001	1.02 [1.00–1.03]
Number of antibiotics [median, IQR]	2 (3)	4 (4)	<0.001	1.27 [1.14–1.40]
Days with CVC [median, IQR]	3 (18)	17.50 (40)	<0.001	1.03 [1.02–1.04]
Days with UC [median, IQR]	0 (20)	21.50 (52)	<0.001	1.03 [1.01–1.04]
Glasgow Score [median, IQR]	15 (8)	10 (11)	0.025	0.92 [0.86–0.99]
Number of antibiotics with resistance/strain [median, IQR]	2 (8)	8 (9)	<0.001	1.13 [1.07–1.19]
Mechanical ventilation [*n*, %]	31(33.33)	156 (63.41)	<0.001	0.29 [0.17–0.49]
Urinary catheter [*n*, %]	46 (49.46)	173 (70.33)	<0.001	0.41 [0.25–0.69]
Tracheostomy [*n*, %]	18 (19.35)	97 (39.43)	<0.001	0.37 [0.20–0.68]
Vasopressors [*n*, %]	22 (23.66)	109 (44.31)	<0.001	0.39 [0.22–0.69]
Blood transfusions [*n*, %]	28 (30.11)	132 (53.66)	<0.001	0.37 [0.22–0.64]
Nasogastric nutrition [*n*, %]	33 (35.48)	141 (57.32)	<0.001	0.41 [0.24–0.69]
Mechanical ventilation 0 h [*n*, %]	62 (66.67)	90 (36.58)	<0.001	3.47 [2.04–5.92]
Mechanical ventilation ≥ 96 h [*n*, %]	29 (31.18)	147 (59.75)	<0.001	0.31 [0.18–0.52]
*Proteus mirabilis* [*n*, %]	75 (80.65)	170 (69.11)	0.034	1.86 [1.01–3.48]
*Providencia stuartii* [*n*, %]	18 (18.35)	76 (30.89)		
Association of 2 pathogens [*n*, %]	8 (8.60)	26 (19.57)	0.046	0.43 [0.17–1,07]
HCAI [*n*, %]	30 (32.96)	147 (59.76)	<0.001	0.32 [0.19–0.55]
Bronchial aspirate [*n*, %]	10 (10.75)	68 (27.64)	<0.001	0.32 [0.14–0.67]
Wound swab [*n*, %]	51 (54.84)	75 (30.48)	<0.001	3.63 [2.10–6.32]
Other [*n*, %]	1 (1.07)	17 (6.91)	0.030	0.15 [0.00–0.96]
DTR phenotype [*n*, %]	7 (7.53)	72 (29.27)	<0.001	0.20 [0.07–0.45]
MDR phenotype [*n*, %]	44 (47.31)	168 (68.29)	<0.001	0.42 [0.25–0.70]
XDR phenotype [*n*, %]	14 (15.05)	91 (36.99)	<0.001	0.30 [0.15–0.58]
Aminoglycoside resistance [*n*, %]	12 (12.90)	69 (28.05)	0.0003	0,38 [0.18–0.77]
ESBL [*n*, %]	15 (16.13)	102 (41.46)	<0.001	0.27 [0.14–0.52]
CR [*n*, %]	13 (13.98)	90 (36.59)	<0.001	0.28 [0.14–0.56]
Fluoroquinolone resistance [*n*, %]	27 (29.03)	133 (54.06)	<0.001	0.35 [0.20–0.60]

Legend: N(*n*), number; IQR, interquartile range; CVC, central venous catheter; UC, urinary catheter; ICU, intensive care unit; h, hour; HCAI, healthcare associated infections; DTR, difficult-to-treat resistance; MDR, multi-drug resistant; XDR, eXtensive Drug Resistance; CI, confidence interval; OR, odds ratio; p, the threshold of statistical significance; CR, Carbapenem resistance; ESBL, Extended spectrum beta-lactamase.

**Table 2:** Variables with statistically significant differences among diabetic patients infected with the two species of *Proteeae*.

Variable	Sample of Diabetic Patients Infected with *P. mirabilis* n1 = 75	Sample of Diabetic Patients Infected with *P. stuartii* n2 = 18	*p* Value
Number of antibiotics [median, IQR]	2 (2)	5 (3)	<0.001
Days with CVC [median, IQR]	2 (12)	16.50 (20)	0.032
Days with UC [median, IQR]	0 (10)	19 (19)	0.019
Number of antibiotics with resistance/pathogen [median, IQR]	1(4)	9 (5)	<0.001
Anti-biotherapy prior to ICU [*n*, %]	9 (12.00)	8 (44.44)	0.003
Mechanical ventilation [*n*, %]	18 (24.00)	13 (72.22)	<0.001
Urinary catheter [*n*, %]	31 (41.33)	15 (83.33)	0.001
Tracheostomy [*n*, %]	8 (10.67)	10 (55.55)	<0.001
Blood transfusions [*n*, %]	17 (22.67)	11 (61.11)	0.001
Nasogastric nutrition [*n*, %]	18 (24.00)	15 (83.33)	<0.001
Mechanical ventilation 0 h [*n*, %]	57 (76.00)	5 (27.77)	<0.001
Mechanical ventilation ≥ 96 h [*n*, %]	16 (21.33)	13 (72.22)	<0.001
Discharge status - Deceased [*n*, %]	20 (26.67)	9 (50.00)	0.054
HCAI [*n*, %]	17 (22.67)	13 (72.22)	<0.001
Blood culture [*n*, %]	3 (4.00)	6 (33.33)	0.001
Catheter tip [*n*, %]	2 (2.67)	6 (33.33)	<0.001
Wound swab [*n*, %]	48 (64.00)	3 (16.67)	<0.001
WT phenotype [*n*, %]	24 (32.00)	0 (0)	0.005
DTR phenotype [*n*, %]	0 (0)	7 (38.89)	<0.001
MDR phenotype [*n*, %]	26 (34.67)	18 (100)	<0.001
XDR phenotype [*n*, %]	3 (4.00)	11 (61.11)	<0.001
Aminoglycoside resistance [*n*, %]	3 (4.00)	9 (50.00)	<0.001
ESBL [*n*, %]	6 (8.00)	9 (50.00)	<0.001
Carbapenem resistance [*n*, %]	1 (1.33)	12 (66.67)	<0.001
Fluoroquinolone resistance [*n*, %]	10 (13.33)	17 (94.44)	<0.001
Sulphonamide resistance [*n*, %]	41 (54.67)	18 (100)	<0.001

Legend: n, number; IQR, interval between quartiles; CVC, central venous catheter; ICU, intensive care unit; h, hour; HCAI, healthcare associated infections; WT, wild type; DTR, difficult-to-treat resistance; MDR, multi-drug resistant; XDR, eXtensive Drug Resistance; OR, odds ratio; HR, hazard ratio; p, the threshold of statistical significance; ESBL, extended spectrum beta-lactamase.

## Data Availability

Not applicable.

## References

[1] Shah B.R., Hux J.E. (2003). Quantifying the risk of infectious diseases for people with diabetes. Diabetes Care.

[2] Muller L.M.A.J., Gorter K.J., Hak E., Goudzwaard W.L., Schellevis F.G., Hoepelman A.I.M., Rutten G.E.H.M. (2005). Increased risk of common infections in patients with type 1 and type 2 diabetes mellitus. Clin. Infect. Dis..

[3] Boyko E.J., Lipsky B., National Diabetes Data Group (U.S.) (1995). A Infection and Diabetes. Diabetes in America.

[4] Peleg A.Y., Weerarathna T., McCarthy J.S., Davis T.M.E. (2007). Common infections in diabetes: Pathogenesis, management and relationship to glycaemic control. Diabetes. Metab. Res. Rev..

[5] Diaconu L., Serban V. (2011). Tratat Roman de Boli Metabolice.

[6] Lipsky B.A., Berendt A.R., Embil J., de Lalla F. (2004). Diagnosing and treating diabetic foot infections. Diabetes. Metab. Res. Rev..

[7] Akash M.S.H., Rehman K., Fiayyaz F., Sabir S., Khurshid M. (2020). Diabetes-associated infections: Development of antimicrobial resistance and possible treatment strategies. Arch. Microbiol..

[8] Alves C., Casqueiro J., Casqueiro J. (2012). Infections in patients with diabetes mellitus: A review of pathogenesis. Indian J. Endocrinol. Metab..

[9] Zhanel G.G., Harding G.K.M., Nicolle L.E. (1990). Asymptomatic bacteriuria in patients with diabetes mellitus. Rev. Infect. Dis..

[10] Nicolle L.E. (2000). Asymptomatic bacteriuria in diabetic women. Diabetes Care.

[11] Dryden M., Baguneid M., Eckmann C., Corman S., Stephens J., Solem C., Li J., Charbonneau C., Baillon-Plot N., Haider S. (2015). Pathophysiology and burden of infection in patients with diabetes mellitus and peripheral vascular disease: Focus on skin and soft-tissue infections. Clin. Microbiol. Infect..

[12] Martin E.T., Kaye K.S., Knott C., Nguyen H., Santarossa M., Evans R., Bertran E., Jaber L. (2016). Diabetes and risk of surgical site infection: A systematic review and meta-analysis. Infect. Control Hosp. Epidemiol..

[13] Siracuse J.J., Nandivada P., Giles K.A., Hamdan A.D., Wyers M.C., Chaikof E.L., Pomposelli F.B., Schermerhorn M.L. (2013). Prosthetic graft infections involving the femoral artery. J. Vasc. Surg..

[14] Arrellano-Valdez F., Urrutia-Osorio M., Arroyo C., Soto-Vega E. (2014). A comprehensive review of urologic complications in patients with diabetes. J. Korean Phys. Soc..

[15] Rus M., Licker M., Musuroi C., Seclaman E., Muntean D., Cirlea N., Tamas A., Vulpie S., Horhat F.G., Baditoiu L. (2020). Distribution of NDM1 carbapenemase-producing proteeae strains on high-risk hospital wards. Infect. Drug Resist..

[16] Rakov C., Porat S.B., Alkalay-Oren S., Yerushalmy O., Abdalrhman M., Gronovich N., Huang L., Pride D., Coppenhagen-Glazer S., Nir-Paz R. (2021). Targeting biofilm of MDR Providencia stuartii by phages using a catheter model. Antibiotics.

[17] Kurmasheva N., Vorobiev V., Sharipova M., Efremova T., Mardanova A. (2018). The Potential Virulence Factors of Providencia stuartii: Motility, Adherence, and Invasion. Biomed. Res. Int..

[18] Musuroi C., Licker M., Rus M., Seclaman E., Muntean D., Vulpie S., Baditoiu L. (2021). Difficult to Treat Proteeae strains in high risk Romanian hospital departments. Rev. Rom. Med. Lab..

[19] Molnár S., Flonta M.M.M., Almaş A., Buzea M., Licker M., Rus M., Földes A., Székely E. Dissemination of NDM-1 carbapenemase-producer Providencia stuartii strains in Romanian hospitals: A multicentre study. J. Hosp. Infect..

[20] Székely E., Damjanova I., Jánvári L., Vas K.E., Molnár S., Bilca D.V., Lorinczi L.K., Tóth Á. (2013). First description of blaNDM-1, blaOXA-48, blaOXA-181 producing Enterobacteriaceae strains in Romania. Int. J. Med. Microbiol..

[21] Axente C., Licker M., Moldovan R., Hogea E., Muntean D., Horhat F., Bedreag O., Sandesc D., Papurica M., Dugaesescu D. (2017). Antimicrobial consumption, costs and resistance patterns: A two year prospective study in a Romanian intensive care unit. BMC Infect. Dis..

[22] Muntean D., Horhat F.G., Badiţoiu L., Dumitraşcu V., Bagiu I.C., Horhat D.I., Coşniţă D.A., Krasta A., Dugăeşescu D., Licker M. (2018). Multidrug-resistant gram-negative bacilli: A retrospective study of trends in a tertiary healthcare unit. Medicina.

[23] Muntean D., Licker M., Horhat F., Dumitrașcu V., Săndesc D., Bedreag O., Dugăeșescu D., Coșniță D.A., Krasta A., Bădițoiu L. (2018). Extensively drug-resistant Acinetobacter baumannii and proteeae association in a Romanian intensive care unit: Risk factors for acquisition. Infect. Drug Resist..

[24] (2018). Commission Implementing Decision (EU) 2018/945 of 22 June 2018 on the communicable diseases and related special health issues to be covered by epidemiological surveillance as well as relevant case definitions. Off J. Eur. Union..

[25] Magiorakos A.P., Srinivasan A., Carey R.B., Carmeli Y., Falagas M.E., Giske C.G., Harbarth S., Hindler J.F., Kahlmeter G., Olsson-Liljequist B. (2012). Multidrug-resistant, extensively drug-resistant and pandrug-resistant bacteria: An international expert proposal for interim standard definitions for acquired resistance. Clin. Microbiol. Infect..

[26] Gajdács M., Bátori Z., Ábrók M., Lázár A., Burián K. (2020). Characterization of Resistance in Gram-Negative Urinary Isolates Using Existing and Novel Indicators of Clinical Relevance: A 10-Year Data Analysis. Life.

[27] McDonnell A., Rex J.H., Goossens H., Bonten M., Fowler V.G., Dane A. (2016). Efficient Delivery of Investigational Antibacterial Agents via Sustainable Clinical Trial Networks. Clin. Infect. Dis. Off. Publ. Infect. Dis. Soc. Am..

[28] Kadri S.S., Adjemian J., Lai Y.L., Spaulding A.B., Ricotta E., Rebecca Prevots D., Palmore T.N., Rhee C., Klompas M., Dekker J.P. (2018). Difficult-to-treat resistance in gram-negative bacteremia at 173 US hospitals: Retrospective cohort analysis of prevalence, predictors, and outcome of resistance to all first-line agents. Clin. Infect. Dis..

[29] Katz D.E., Friedman N.D., Ostrovski E., Ravid D., Amrami N., Avivi D., Mengesha B., Zaidenstein R., Lazarovitch T., Dadon M. (2016). Diabetic foot infection in hospitalized adults. J. Infect. Chemother..

[30] Al Benwan K., Al Mulla A., Rotimi V.O. (2012). A study of the microbiology of diabetic foot infections in a teaching hospital in Kuwait. J. Infect. Public Health.

[31] Saseedharan S., Sahu M., Chaddha R., Pathrose E., Bal A., Bhalekar P., Sekar P., Krishnan P. (2018). Epidemiology of diabetic foot infections in a reference tertiary hospital in India. Braz. J. Microbiol..

[32] Heravi F.S., Zakrzewski M., Vickery K., Armstrong D.G., Hu H. (2019). Bacterial diversity of diabetic foot ulcers: Current status and future prospectives. J. Clin. Med..

[33] Laupland K.B., Parkins M.D., Ross T., Pitout J.D.D. (2007). Population-based laboratory surveillance for tribe proteeae isolates in a large Canadian health region. Clin. Microbiol. Infect..

[34] Al-Rubeaan K.A., Moharram O., Al-Naqeb D., Hassan A., Rafiullah M.R.M. (2013). Prevalence of urinary tract infection and risk factors among Saudi patients with diabetes. World J. Urol..

[35] Chiţă T., Timar B., Muntean D., Bădiţoiu L., Horhat F., Hogea E., Moldovan R., Timar R., Licker M. (2017). Urinary tract infections in romanian patients with diabetes: Prevalence, etiology, and risk factors. Ther. Clin. Risk Manag..

[36] Jha P., Baral R., Khanal B. (2014). Prevalence of Uropathogens in Diabetic Patients and Their Susceptibility Pattern at a Tertiary Care Center in Nepal-A Retrospective Study. Int. J. Bio. Lab. Sci..

[37] Gautam S., Sapkota R. (2020). Comparative Study of Isolates Associated With Urinary Tract Infection among Diabetic and Non-Diabetic Patients Attending Tertiary Care Hospital, Chitwan, Nepa. Int. J. Innov. Sci. Res..

[38] Warren J.W. (1986). Providencia stuartii: A common cause of antibiotic-resistant bacteriuria in patients with long-term indwelling catheters. Rev. Infect. Dis..

[39] Wie S.H. (2015). Clinical significance of providencia bacteremia or bacteriuria. Korean J. Intern. Med..

[40] Sabri S.M., Rizk M.S., Badr M.F.- (2012). ESBL-Producing Multidrug-Resistant Providencia Stuartii Infection in Mansoura University Hospitals. Egypt. J. Med. Microbiol..

[41] Darouiche R.O. (2001). Device-associated infections: A macroproblem that starts with microadherence. Clin. Infect. Dis..

[42] Choi H.K., Kim Y.K., Kim H.Y., Park J.E., Uh Y. (2015). Clinical and microbiological features of providencia bacteremia: Experience at a tertiary care hospital. Korean J. Intern. Med..

[43] Tumbarello M., Citton R., Spanu T., Sanguinetti M., Romano L., Fadda G., Cauda R. (2004). ESBL-producing multidrug-resistant Providencia stuartii infections in a university hospital. J. Antimicrob. Chemother..

[44] Hayakawa K., Marchaim D., Divine G.W., Pogue J.M., Kumar S., Lephart P., Risko K., Sobel J.D., Kaye K.S. (2012). Growing prevalence of Providencia stuartii associated with the increased usage of colistin at a tertiary health care center. Int. J. Infect. Dis..

[45] Abdallah M., Balshi A. (2018). First literature review of carbapenem-resistant Providencia. New Microbes New Infect..

